# Glucose regulation and association with Vitamin D and parathyroid hormone – differences across Middle Eastern and Caucasian ethnicities

**DOI:** 10.1007/s40200-024-01543-y

**Published:** 2024-12-19

**Authors:** Nadine Fadhel Dhaher, Per Wändell, Louise Bennet

**Affiliations:** 1 Department of Clinical Sciences in Malmö, Lund University, Lund, Sweden; 2 Department of Endocrinology, Skåne University Hospital, Malmö, Sweden; 3 Division of Family Medicine and Primary Care, Department of Neurobiology, Care Sciences and Society, Karolinska Institute, Huddinge, Sweden; 4 Center for Primary Health Care Research, Lund University, Malmö, Sweden; 5 Clinical Trial Unit, Clinical Studies Sweden – Forum South, Lund University Hospital, Lund, Sweden

**Keywords:** Insulin action, Insulin secretion, Vitamin D, Parathyroid hormone, Middle East, Migration

## Abstract

**Background:**

Middle Eastern (ME) immigrants to Europe have a heavy burden of metabolic disorders including a higher prevalence of insulin resistance, T2D and obesity as compared to native-born Europeans. Vitamin D insufficiency and deficiency are prevalent conditions in people originating from the ME.

**Aims:**

To study the differences in the levels of 25(OH)D and parathyroid hormone (PTH) across ME and European ethnicity, and the effect of 25(OH)D and PTH on insulin action and secretion.

**Methods:**

Vitamin D and PTH levels were assessed in a population-based cohort of 918 participants (449 Swedes and 469 Iraqis) aged 30–75 years. The differences between the groups in the adjusted levels of Vitamin D and PTH were studied using multiple regression analysis. Differences in insulin action and secretion, in relation to risk markers including Vitamin D and PTH, were assessed using multiple regression analysis.

**Results:**

Vitamin D and PTH adjusted levels differed significantly between the groups; 92% of the Iraqi-born versus 45% of the Swedish-born individuals had Vitamin D levels below 50 nmol/L. The mean levels of PTH (SD) were higher in Iraqi-born compared to native Swedish-born (5.1 (2.3) vs. 3.8 (1.6) pmol/L, p = < 0.001). Insulin sensitivity was lower in Iraqi-born (79.16 vs. 98.97, β ^-^0.085, 95% CI ^−.1^63 to ^−.0^07) but after adjustment for the confounding effect of Vitamin D, the differences in insulin action observed between the groups were no longer significant.

**Conclusion:**

The ethnic differences in insulin action could be explained by differences in the levels of Vitamin D.

## Background

A total of 20% of the population of Sweden today is comprised of individuals born abroad with the largest immigrant groups originating from the Middle East (ME) [1]. ME immigrants to Europe often have a heavy burden of metabolic disorders including a higher prevalence of type 2 diabetes (T2D), obesity as well as family history of diabetes compared to Swedish- born individuals [2].

25-hydroxyvitamin D (25(OH)D) insufficiency i.e. *mild* (serum 25(OH)D < 50 and ≥ 25 nmol/L), *moderate* (serum 25(OH)D < 25 and ≥ 12.5 nmol/L) and *severe* deficiency (< 12.5 nmol/L) [3] are prevalent conditions globally and some of the lowest levels are reported from the ME region [4].

Vitamin D, which is also known as calciferol, exists in two major forms; vitamin D_2_ (ergocalciferol) which is human-made and added to foods, and D_3_ (cholecalciferol) which is synthesised in the skin from 7-dehydrocholesterol and consumed from animal-based foods [5, 6]. Both forms are synthesised commercially and found in fortified food and differ only in their side chain structure [5, 6]. After becoming biologically active through two enzymatic hydroxylation reactions both forms have identical effects in the body [6]. The renal activation of 25(OH)D is up-regulated by the parathyroid hormone (PTH) and down-regulated via fibroblast-like growth factor (FGF23) [7]. Following activation, calcitriol exerts its action at a transcriptional level, regulating gene expression after binding to its receptors in the nuclei of target cells - Vitamin D receptors (VDRs) [8]. The classical actions of 25(OH)D involve regulating serum calcium and phosphate, which maintains bone homeostasis. Potential mechanisms on how vitamin D affects glucose homeostasis include the identification of specific VDRs on both human and rat pancreatic β-cells [9], the expression of 1-⍺-hydroxylase in rat pancreatic β-cells [10], and the presence of a vitamin D response element in the human insulin gene promoter [11].

PTH has also been found to be inversely related to insulin sensitivity [12] and higher levels are associated with insulin resistance and higher T2D prevalence [13]. There is an inverse relationship between serum 25-hydroxyvitamin D (25-OHD) and serum PTH; at low serum calcium levels, PTH mobilises calcium stores and increases calcium absorption and reabsorption. 25(OH)D and calcium exert negative feedback on the parathyroid glands to inhibit the production and release of PTH [14, 15].

Reports from the MEDIM (impact of Migration and Ethnicity on Diabetes in Malmö) study showed that Iraqi-born immigrants have twice as high T2D prevalence compared to the Swedish-born control group, higher insulin resistance and a higher prevalence of T2D-related risk factors including obesity and dyslipidaemia [2]. The fact that ME immigrants share the same risk factors for T2D, and 25(OH)D deficiency is perhaps not only a coincidence, raises the question about potential significant ethnic differences in the associations between 25(OH)D, PTH and glucose regulation. Our aims were to study the differences in levels of 25(OH)D and PTH between Iraqi-born and Swedish-born individuals and investigate potential interactions across ethnicity in the effect of 25(OH)D and PTH on insulin action and secretion.

## Materials and methods

Iraqi or Swedish-born residents of Malmö, Sweden aged 30–75 years old, were invited to participate in the population-based MEDIM study as previously described [2]. This study was conducted between February 2010 to December 2011. All oral as well as written information throughout the study were provided in participants native language i.e. Arabic and/or Swedish. People suffering from severe physical or mental illness, pregnancy or having a diagnosis of type one diabetes were not included in the study. The participation rate for the Iraqis was 64% and it was 40% for the Swedish-born. The age and sex distribution of the Iraqi participants did not differ compared to the eligible background population. All participants were recruited from the same socioeconomic area as the Swedish-born as the control group, matched for sex and age. All participants oral and written information about the study and signed informed consent prior to participation. A health examination was conducted including anthropometrics, fasting blood samples and an oral glucose tolerance test (OGTT). Due to ethical considerations we did not want to expose people with known Type 2 diabetes to hyperglycemia, thus they did not undergo the OGTT [16]. Sociodemographic and lifestyle behaviour data were collected using self-administered questionnaires in Swedish or Arabic [17]. The questionnaires also included questions regarding dietary habits according to the Nordic Nutrition recommendations [18]. In the dietary habits we included the intake of fish and shrimp as a main dish as a source of 25(OH)D. The participants were asked to provide a medication list with information regarding previous and current diagnoses [19].

A flowchart describing the recruitment of MEDIM participants is attached (Supplement 1). Participants were examined in parallel throughout the study period. 25(OH)D and PTH were analysed in participants recruited from the start of the study (February 2010) until December 2011 (the original study ended in Dec 2012) to avoid the influence of seasonal variation. 25(OH)D and PTH were analysed in a total of 918 participants (469 Iraqis and 449 Swedes).

A HemoCue photometer (HemoCue AB, Ängelholm, Sweden) was used to measure plasma glucose levels, fasting reference **≥** 7.0 mmol/l, taken twice capillary, non-fasting capillary 2 h glucose ≥ 12.2 mmol/l were the criteria for a diabetes diagnosis [20]. Serum concentrations of 25(OH)D were assessed by a chemiluminescence immunoassay and expressed in nmol/L. Radioimmunoassay (Access© Ultrasensitive Insulin, Beckman Coulter, USA) and high-pressure liquid chromatography (Bio-Rad) were used to estimate serum insulin levels and HbA_1C_ [20]. PTH was analysed using the Atellica IM PTH-method and measured in pmol/L, ref: 2,0–8,5 pmol/L.

### Definitions

Insulin sensitivity index (ISI), insulin secretion (corrected insulin response, CIR) and DIo were assessed through insulin and glucose measured at 0, 30 min, 60 min and 120 min intervals during OGTT (Matsuda indices) [16, 21, 22]. Participants with T2D did not undergo an OGTT.

ISI, insulin sensitivity index, by Matsuda provides an estimate of hepatic and muscle insulin sensitivity [23]. CIR is assessed to measure glucose-stimulated insulin secretion and provides an estimate of beta-cell function. DIo is an estimate of beta-cell function adjusted for insulin resistance. DIo is the product of CIR and ISI. ISI, CIR and Dio were calculated from the OGTT using Matsuda indices [16, 21, 23, 24].

### Statistical analysis

Statistical analyses were performed using IBM SPSS version 29.0. Data are presented in means (standard deviation, SD), numbers (percentages) or for skewed data, medians, and a p-value of < 0.05 was considered statistically significant.

Multiple linear regression models were used to explore the associations between insulin secretion, insulin sensitivity (dependent variables) and other covariates as independent variables. Multiple linear regression models were also used to study the differences in 25(OH)D and PTH between the groups, adjusting for the confounding effects of relevant variables (country of origin, age, sex, BMI, dietary habits, and physical activity). Skewed variables were ln - transformed before regression analysis to approximate normal distributions.

## Results

In total, the levels of 25(OH)D and PTH were analysed in 918 men and women (449 Swedes and 469 Iraqis), ranging from 30 to 75 years of age, participated in the study.

Table 1 shows the basic- clinical- as well as biochemical characteristics of both groups; the Iraqi immigrants were younger, had higher BMI, were less physically active and had higher prevalence of T2D. Furthermore, they presented with a heavy burden of family history of T2D, higher fasting insulin levels, lower insulin sensitivity but also lower alcohol consumption.

**Table 1:** Characteristics of study participants Iraqi and Swedish-born living in Malmö

Variable	Country of birth		*P*-value
	Iraq (*N* = 469)	Sweden (*N* = 450)	
Age (years)	48.1 (9.6)	49.7 (11.0)	0.018
Male sex, n (%)	279 (59.5)	236 (52.4)	0.033
Body mass index, kg/m^2^	28.9 (4.3)	27.1 (4.3)	< 0.001
Waist circumference, (cm)	97.1 (11.4)	94.5 (13.2)	0.002
PA h/week	2.3 (2.5)	4.1 (2.5)	< 0.001
Total cholesterol (mmol/L)	4.9 (1.0)	5.2 (1.0)	< 0.001
p-LDL (mmol/L)	3.2 (0.9)	3.3 (0.9)	0.097
p-HDL (mmol/L)	1.1 (0.3)	1.4 (0.4)	< 0.001
p-Triglycerides (mmol/L)	1.6 (1.0)	1.2 (0.8)	< 0.001
Smokers, *n* (%)	121 (25.8)	118 (26.2)	0.88
Blood pressure	130/80 (19/12)	136/83 (20/12)	< 0.001
Family history of T2D, *n* (%)	144 (30.7)	108 (24)	< 0.001
ISI (mmol/L*mIE/L − 1) ^1^	79.16	98.97	< 0.001
CIR (mmol/L*mmol/L*mIE/L − 1) ^1^	170.0	143.4	0.02
DIo(mmol/L*mmol/L*mmol/L) ^1^	13,245.0	14,473.0	0.30
HbA1c (mmol/mol)	37.7 (10.6)	36.1 (8.5)	0.014
Fasting glucose, (mmol/L)	5.9 (1.7)	5.7 (1.3)	0.050
Fasting insulin^1^	9.0	7.0	< 0.001
Diabetes	56 (11.9)	26 (5.8)	0.001
Alcohol	85 (18.1)	365 (81.1)	< 0.001
Fish/shrimp as main dish, (times/week)	3.25 (0.8)	3.04 (0.9)	< 0.001

Crude data are presented as means (SD) or as numbers (percentages); family history refers to parents, children and/or siblings; LDL/HDL is low-density/high-density lipoprotein. ^1^Differences in medians between the groups

Table 2 shows the differences in the levels of 25(OH)D and PTH between the groups related to sex. In total, 22.3% of the Iraqi-born participants had 25(OH)D levels < 25nmol/L, but only 7.7% of this group had elevated PTH values > 8.5pmol/L.

**Table 2:** Descriptives of 25(OH)D and PTH levels among the participants

Variable	Country of birth		*P*-value
	Iraq (*N* = 469)	Sweden (*N* = 450)	
25(OH)D^1^ nmol/L	29 (25, 35)	52 (40, 63)	< 0.001
25(OH)D^1^ male sex	29 (25, 35)	51 (39, 62)	< 0.001
25(OH)D^1^ female sex	28 (25, 34)	53 (41, 64)	< 0.001
25(OH)D < 25 nmol/L (%)	105 (22.3)	7 (1.6)	< 0.001
25(OH)D < 50 nmol/L (%)	432 (92.1)	204 (45.3)	< 0.001
PTH (SD) pmol/L	5.1 (2.3)	3.8 (1.6)	< 0.001
PTH (SD), male sex	5.3 (2.9)	4.0 (1.5)	< 0.001
PTH (SD), female sex	4.8 (2.1)	3.6 (1.6)	< 0.001
PTH > 8.5 pmol/L (%)	36 (7.7)	6 (1.3)	< 0.001

Crude data are presented as means (SD) or as numbers (percentages); ^1^Differences in medians between the groups (IQR)

The differences in insulin action and secretion, in relation to risk markers including 25(OH)D and PTH, are displayed in Table 3 and Supplementary Table 2. Table 3 shows significant differences in ISI between the groups but in Model 5, the differences in ISI were no longer significant when adjusted for 25(OH)D. No differences in insulin secretion were observed between the groups (Supplementary Table 2).

**Table 3:** Linear regression models with ln ISI as a dependent factor expressed as β coefficients with 95% confidence intervals

Variable	Model 1 *N* = 831 *R*^2^ = 0.032	Model 2 *N* = 831 *R*^2^ = 0.297	Model 3 *N* = 808 *R*^2^ = 0.299	Model 4 *N* = 808 *R*^2^ = 0.545	Model 5 *N* = 808 *R*^2^ = 0.300
Born in Sweden Born in Iraq	Reference ^−^0.222*** ^−^0.305 to ^−^0.139	Reference ^−^0.089* ^−^0.162 to ^−^0.016	Reference ^−^0.085* ^−^0.160 to ^−^0.010	Reference ^−^0.085* ^−^0.163 to ^−^0.007	Reference ^−^0.058 ^−^0.150 to 0.033
Age (years)		− 0.001 − 0.004 to 0.003	− 0.001 − 0.004 to 0.003	^−^0.001 ^−^0.004 to 0.003	^−^0.001 ^−^0.005 to 0.003
Female sex Male sex		^−^0.225*** ^−^0.297 to ^−^0.153	^−^0.229*** ^−^0.302 to ^−^0.155	^−^0.229*** ^−^0.303 to ^−^0.155	^−^0.227*** ^−^0.301 to ^−^0.153
Body mass index (kg/m^2^)		^−^0.068*** ^−^0.076 to ^−^0.060	^−^0.069*** ^−^0.077 to ^−^0.060	^−^0.069*** ^−^0.077 to ^−^0.060	^−^0.068*** ^−^0.077 to ^−^0.060
Diet^			0.016 ^−^0.026 to 0.058	0.016 ^−^0.026 to 0.058	0.021 ^−^0.022 to 0.063
p-PTH				0.00 ^−^0.019 to 0.019	0.002 ^−^0.017 to 0.022
25(OH)D					0.002 ^−^0.001 to 0.004

*P* < 0.05*, *P* < 0.01**, *P* < 0.001***, ^ Fish/shrimp as main dish, (times/week)

**Fig. 1:**
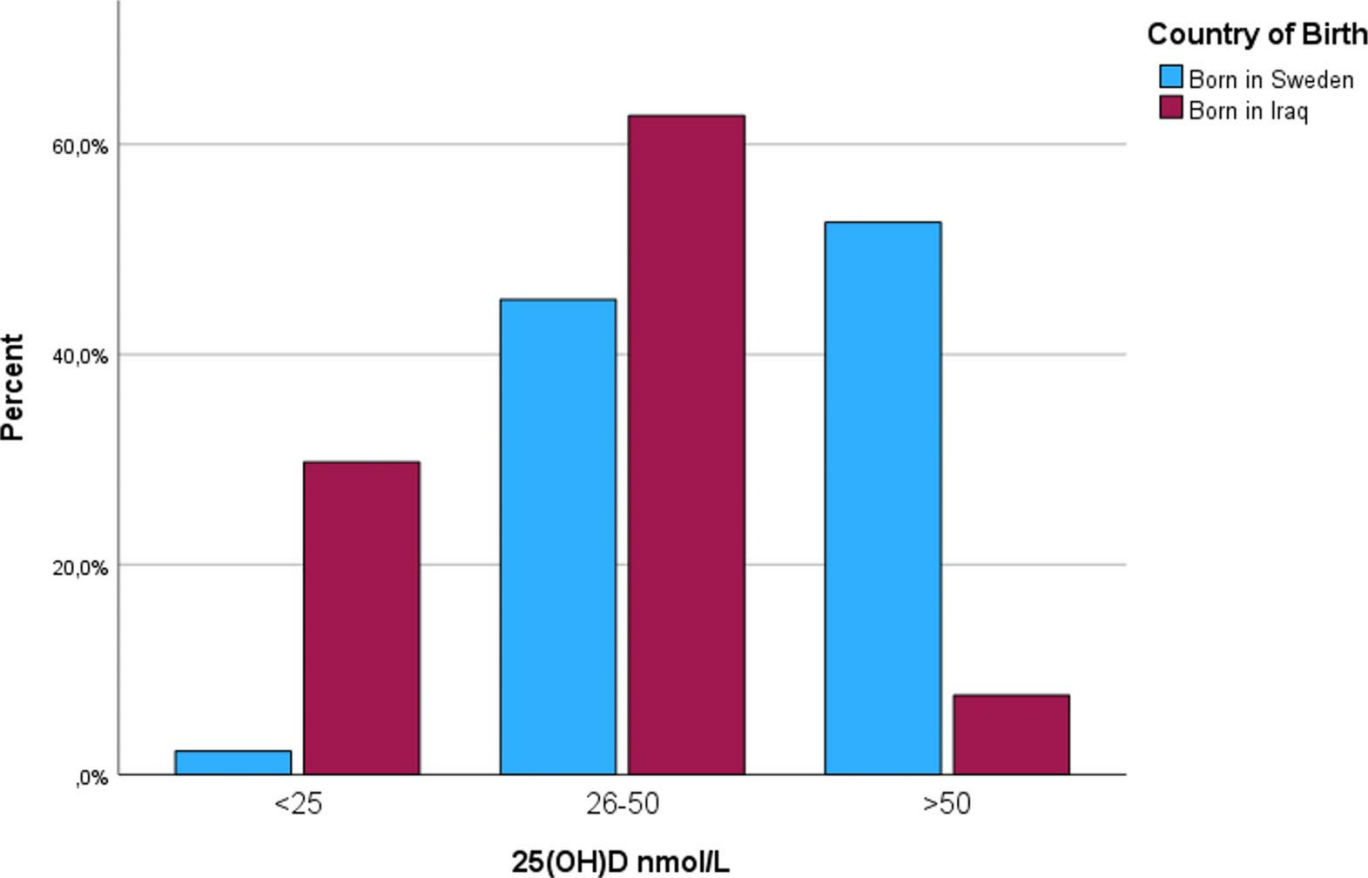
Distribution of 25(OH)D levels based on the country of origin.

**Fig. 2:**
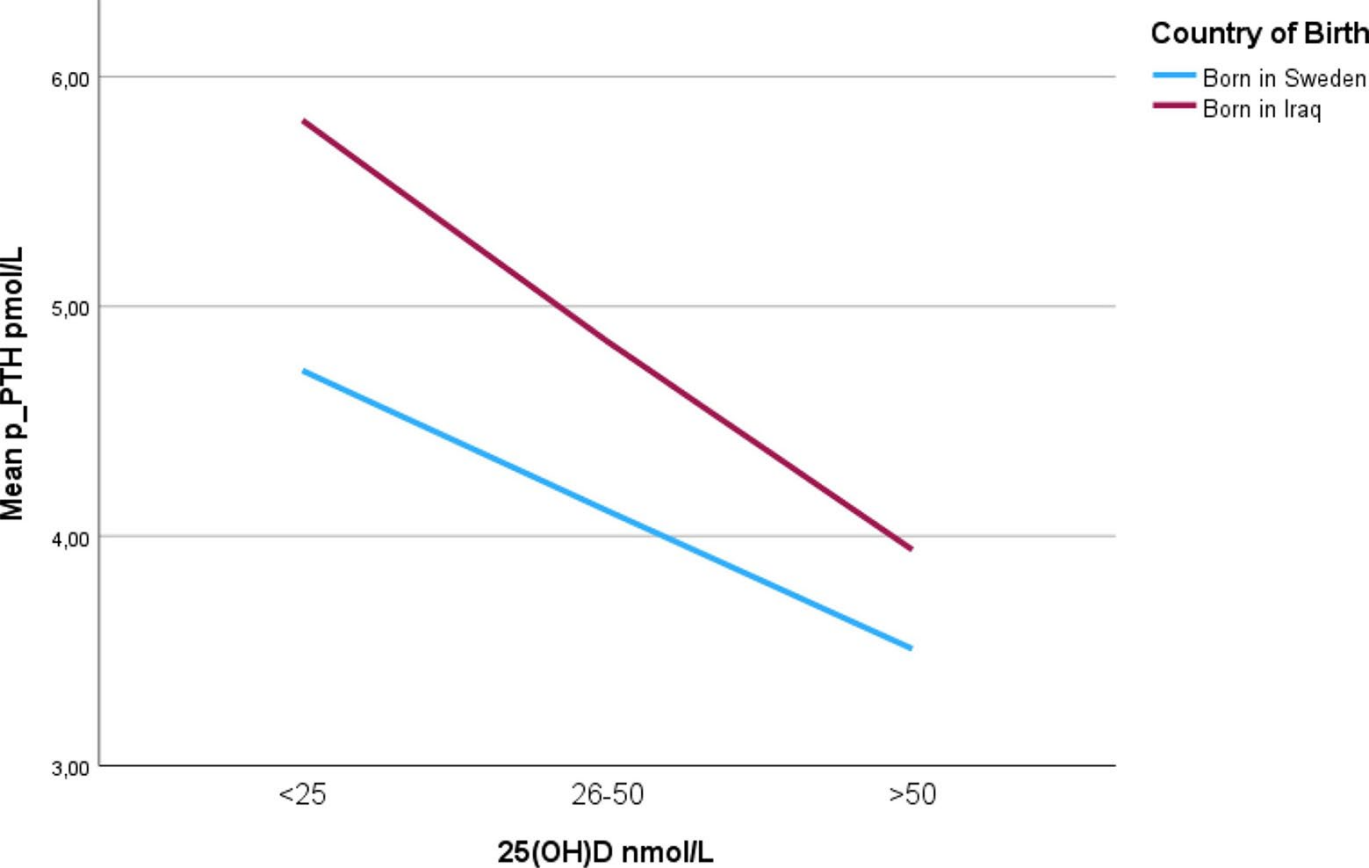
The correlation between PTH and 25(OH)D in based on the country of origin.

Figure 1 illustrates the distribution of the 25(OH)D levels based on the country of origin, with dominance of the Iraqi group in the lower 25(OH)D categories. In Fig. 2, the correlation between p-PTH and 25(OH)D is illustrated, the inverse proportionality between PTH and 25(OH)D is observed, with higher p-PTH levels in the Iraqi group.

Tables 4 and 5 show the adjusted differences between the levels of 25(OH)D and PTH between the groups. 25(OH)D as well as PTH levels differed significantly between the groups and remained in the fully adjusted Model 4 in Tables 4 and 5, with the Iraqi-born participants having significantly lower levels of 25(OH)D and higher levels of PTH. The differences in 25(OH)D between the groups remained significant even taking into consideration the duration of time spent in Sweden for the Iraqi-born individuals (β for the effect of time spent in Sweden on 25(OH)D: ^-^0.004, 95% CI ^-^0.012 to 0.005, *P* = 0.399), adjustment to age and sex was made.

**Table 4:** Linear regression models with 25(OH)D as a dependent factor expressed as β coefficients with 95% confidence intervals

Variable	Model 1 *N* = 917 *R*^2^ = 0.379	Model 2 *N* = 916 *R*^2^ = 0.395	Model 3 *N* = 916 *R*^2^ = 0.442	Model 4 *N* = 739 *R*^2^ = 0.510
Born in Sweden Born in Iraq	Reference ^−^0.487*** ^−^0.527 to ^−^0.446	Reference ^−^0.464*** ^−^0.505 to ^−^0.423	Reference ^−^0.414*** ^−^0.455 to − 0.373	Reference ^−^0.389*** ^−^0.434 to ^−^0.343
Age (years)		0.004*** 0.002 to 0.006	0.004*** 0.002 to 0.005	0.005*** 0.003 to 0.007
Female sex Male sex		^−^0.035 ^−^0.076 to 0.005	^−^0.017 ^−^0.056 to 0.022	0.000 ^−^0.041 to 0.041
Body mass index (kg/m^2^)		^−^0.007** ^−^0.012 to ^−^0.003	^−^0.005* ^−^0.009 to 0.000	^−^0.005* ^−^0.010 to ^−^0.001
PTH			^−^0.044*** ^−^0.054 to ^−^0.034	^−^0.043*** ^−^0.054 to ^−^0.033
PA h/week				0.012* 0.004 to 0.020
Diet^				^−^0.061*** ^−^0.084 to ^−^0.037

*P* < 0.05*, *P* < 0.01**, *P* < 0.001***, ^ Fish/shrimps as main dish, (times/week)

**Table 5:** Linear regression models with PTH as a dependent factor expressed as β coefficients with 95% confidence intervals

Variable	Model 1 *N* = 917 *R*^2^ = 0.097	Model 2 *N* = 916 *R*^2^ = 0.123	Model 3 *N* = 916 *R*^2^ = 0.177	Model 4 *N* = 739 *R*^2^ = 0.216
Born in Sweden Born in Iraq	Reference 1.293*** 1.038 to 1.549	Reference 1.141*** 0.881 to 1.401	Reference 0.480** 0.178 to 0.783	Reference 0.543* 0.198 to 0.889
Age (years)		^−^0.007 ^−^0.019 to 0.005	^−^0.001 ^−^0.013 to 0.011	0.013* 0.000 to 0.026
Female sex Male sex		0.425** 0.170 to 0.680	0.358* 0.109 to 0.606	233 ^−^0.043 to 0.508
Body mass index (kg/m^2^)		0.057*** 0.029 to 0.086	0.045** 0.017 to 0.073	0.054*** 0.023 to 0.086
25(OH)D			^−^0.035*** ^−^0.044 to ^−^0.026	^−^0.037*** ^−^0.047 to ^−^0.026
PA h/week				^−^0.019 ^−^0.074 to 0.035
Diet^				0.080 ^−^0.079 to 0.238

*P* < 0.05*, *P* < 0.01**, *P* < 0.001***, ^ Fish/shrimps as main dish, (times/week)

## Discussion

Our data shows significant differences in the levels of 25(OH)D and PTH between the Iraqi- born immigrants and the Swedish-born natives, which remained significant after adjusting for the confounding effect of factors influencing vitamin D and PTH.

Individuals with T2D and obesity have been shown - compared to healthy individuals - to have significantly lower levels of 25(OH)D [5]. Epidemiological studies, such as The Longitudinal Aging Study Amsterdam [25], the 1958 British Birth Cohort [25] and the Nurse’s Health Study [26], have found 25(OH)D deficiency to be associated with higher fasting glucose, insulin resistance and higher relative risk for T2D [27].

Significant associations have been found between 25(OH)D deficiency and insulin resistance and T2D [28] and, in the earlier publications from the MEDIM population study, significant ethnic differences in insulin action were reported between Iraqi-born immigrants to Sweden and Swedish-born natives, after adjusting for the confounding effect of age, BMI, sex, family history and lifestyle habits [2, 23]. In this study, differences in insulin action between Iraqi and Swedish-born participants diminished after adjusting for the confounding effect of differences in 25(OH)D (Table 3, model 5). Ethnic differences in insulin sensitivity associated with 25(OH)D levels have been reported in other cross-sectional studies [29, 30]. A meta-analysis, which included 18 RCTs and 20 observational studies with 1243 and 11,063 participants with diabetes, showed significantly higher insulin sensitivity among participants with vitamin D supplementation thus suggesting that supplementation should be integrated into conventional medical approaches for prevention and treatment of T2D [31–33]. The studies in the meta-analysis included different ethnicities but no subgroup analyses were conducted. However, intervention studies where vitamin D supplementation was used have not been able to report improvements in insulin sensitivity among subjects with prediabetes, overweight and obesity in people of different ethnicities [34, 35]. Authors suggest that the inconsistency in treatment results could be explained by the short duration of treatment, the use of lower doses and variability in participants’ vitamin D-deficiency status [35].

The reported associations between the levels of 25(OH)D and insulin secretion are contradictory [36] and, in our study, we do not show such an association. Other studies suggest that 25(OH)D exerts a positive effect on insulin secretion only when calcium levels are adequate [37]. Calcium levels are not analysed in our cohort and therefore future studies are needed to better understand the correlation between 25(OH)D deficiency and insulin secretion.

Vitamin D deficiency causes rickets in children and osteopenia, osteoporosis, and fractures in adults [38]. Our findings of a higher prevalence of 25(OH)D deficiency in ME immigrants are consistent with earlier studies and thus our results are not only specific to an Iraqi immigrant population but to the ME population in general [4, 39–41]. Food and lifestyle habits among first-generation immigrants are considered to correspond with the cultural habits in their country of origin with little adaptation to the host country, which provides further generalisability to our study findings [42]. When comparing the results from 41 observational studies from the ME and North Africa, the prevalence of vitamin D insufficiency ranged between 44 and 96% in adults and 12–96% in children [4]. 25(OH)D insufficiency/deficiency is more common in non-western immigrants to Europe [43–45]. A Norwegian study including 491 and 509 immigrant men and women from the ME and North Africa respectively, showed that the median 25(OH)D level was 28 nmol/L and the prevalence of vitamin D deficiency (< 25 nmol/L) was 37.2% [43].

In a recent Swedish study by Taloyan et al. [41], 25(OH)D levels among 232 participants of ME descent without a diagnosis of T2D were compared to 278 participants of Swedish descent and showed that those of ME descent had almost seven times higher odds of having inadequate 25(OH)D levels, defined as 25(OH)D levels below 50 nmol/L. There was also a trend towards higher fasting blood sugar among individuals of ME ancestry. The participants were all recruited when seeking medical attention in primary health care units and as such are not a representative sample of a healthy migrant group. The participants were not asked to provide information about dietary habits and physical activity.

The highly prevalent 25(OH)D deficiency in non-western immigrants could be explained by lower exposure to sunshine among ME immigrants, due to the excessive sunshine in their countries of origin [40]. Other plausible contributing factors could be the differences in skin pigmentation and the modest clothing style among ME immigrants due to religious causes or cultural habits [40, 46], as well as lack of dairy products in the ME diet [46]. However, in our study, the samples for 25(OH)D and PTH analysis were collected during the same period for both Iraqi and Swedish-born participants in a parallel manner to avoid seasonal variation. Despite the Iraqi males having a dress code similar to that of Swedish-born males, we still saw significantly lower levels of 25(OH)D among Iraqi males. In a UK study by Smith et al. [47], vitamin D levels in Bangladeshi migrants were very low with 94% being deficient or insufficient (≤ 50 nmol/L). Lower levels of 25(OH)D were associated with increased body mass index.

The inverse relationship between 25(OH)D and PTH has been reported across ethnicities [48, 49] but there is inconsistent evidence regarding the 25(OH)D threshold for which PTH is elevated [49–51].

In a nationwide Swedish study (*n* = 2,775,736) [52], a generally lower risk for osteoporotic fractures in first-generation immigrants was observed with exception of Iraqi men who had a higher osteoporotic fracture risk. Among the second-generation immigrants to Sweden, the osteoporotic fracture risk was similar to Swedish natives [53]. Thus, it is still unclear if the levels of 25(OH)D, considered “adequate” for bone and mineral metabolism in a ME population are the same as in a European population.

Serum 25(OH)D concentrations < 50 nmol/l are generally considered inadequate for bone- and overall health [54]. With this study, we wish to raise awareness among clinicians regarding the high prevalence of vitamin D deficiency among ME immigrants. Randomized controlled trials (RCTs) investigating the potential effect of vitamin D supplementation in improving insulin sensitivity are crucial.

### Strengths and weaknesses of the study

Several previous studies have reported lower 25(OH)D levels in ME individuals. In the current study, not only differences in the levels are reported but we have factored in anthropometrical measures, age, sex, dietary, lifestyle habits as well as glucose metabolism. Blood samples from both groups were collected during the same time frame. The association between insulin action and secretion to the levels of 25(OH)D and PTH are also unique to this study since we are able to consider the effect of other potentially confounding variables, also considering the fact that earlier data have been published from the MEDIM cohort regarding the differences in insulin action and secretion between the groups and now and adjustment to 25(OH)D has been made. The MEDIM study is thoroughly phenotyped and diet habits covered by validated questionnaires - that were feasible to conduct in a population-based setting (i.e. from the Swedish food agency). Sun exposure habits were not covered by the questionnaires. However, since we see no differences in the levels of Vitamin D between Iraqi males and females -even though Arabic females dress in a modest manner- but we see large differences in Vitamin D between Iraqi men and Swedish men -that dress similarly- we conclude that sun exposure does not seem to affect the differences in Vitamin D levels across Middle Eastern and Caucasian ethnicities significantly. A weakness of the study is the lack of information regarding differences in outside activities, and sun exposure between the groups as well as calcium levels that could have contributed to differences in vitamin D levels. Other limitations are the lack of information regarding the intake of Vitamin D supplements, fortified food or Vitamin D rich food products other than the ones listed in this study.

The selection of participants in the MEDIM cohort are representative of the background population regarding the prevalence of self-reported diabetes in participants versus non-participants [2].

### Unanswered questions and future research

Interventional studies comparing the effect of vitamin D supplementation on insulin action and secretion, in ME immigrants and native Europeans, are needed as well as cohort studies addressing the incidence of osteoporosis and fracture risk in relation to the levels of 25(OH)D, PTH and Calcium.

## Conclusion

This study shows significant differences in the levels of 25(OH)D and PTH between a group of Iraqi-born immigrants compared to Swedish-born natives in Malmö, Sweden, with the Iraqis having lower levels of 25(OH)D and higher levels of PTH. The ethnic differences in insulin action previously found between Iraqi-born and native-born Swedes seems to be correlated to differences in 25(OH)D levels but further studies are needed to confirm this relationship.

## Data Availability

The datasets used and/or analysed during the current study are available from the corresponding author upon reasonable request.
